# Integrated multi-omics reveals persistent extracellular matrix remodeling in chronic duck footpad dermatitis

**DOI:** 10.1016/j.psj.2026.107830

**Published:** 2026-09-23

**Authors:** Cui Ma, Yunhe Zong, Hao Zhang, Zhenhua Liang, Ailuan Pan, Jinsong Pi, Yan Wu

**Affiliations:** a Institute of Animal Science and Veterinary Medicine, Hubei Academy of Agricultural Sciences, Wuhan, 430064, China; b Key Laboratory of Animal Embryo Engineering and Molecular Breeding of Hubei Province, Wuhan, 430064, China

**Keywords:** Footpad dermatitis, Extracellular matrix remodeling, Microbiota, Metabolic alterations, Duck

## Abstract

Footpad dermatitis (FPD) is a common welfare and production problem in intensively reared poultry, yet the mechanisms responsible for its chronic progression remain poorly understood. To investigate the biological changes associated with chronic FPD, healthy and FPD-affected ducks (Health and FPD groups) were compared using histopathology, transcriptomics, proteomics, microbiome profiling, and metabolomics, with 3–8 biological replicates per group depending on the analytical platform. Histological examination demonstrated marked extracellular matrix (ECM) remodeling, characterized by collagen disorganization, an altered type I/type III collagen ratio, and disrupted matrix organization. Transcriptomic and proteomic analyses consistently identified alterations in ECM–receptor interaction, focal adhesion, and integrin β1/TGF-β-related signaling pathways, showing host molecular alterations associated with persistent ECM remodeling. FPD was also accompanied by marked alterations in both skin and gut microbial communities together with widespread metabolic alterations. Correlation analysis further identified associations among differential microbial taxa, metabolites, and ECM-related genes and proteins. Collectively, these findings indicate that chronic FPD is characterized by persistent extracellular matrix remodeling accompanied by extensive host molecular, microbial, and metabolic alterations. This study extends the current understanding of chronic FPD and provides a foundation for future studies aimed at improving footpad health, animal welfare, and production sustainability in poultry.

## Introduction

Footpad dermatitis (FPD) is a common welfare problem in intensively reared poultry and is associated with impaired locomotion, reduced production performance, and substantial economic losses. As lesions progress, disruption of extracellular matrix (ECM) organization becomes increasingly evident, accompanied by collagen disorganization and impaired tissue repair (Abdel-Hamid et al., 2020; Qin et al., 2019). Previous studies have associated FPD with prolonged mechanical loading, flooring and environmental conditions, poor litter quality, and microbial colonization (Biesek et al., 2024; Liu et al., 2023; Piller et al., 2020). However, these factors do not fully explain the persistence of tissue injury or the biological processes underlying ECM remodeling during chronic disease progression. A clearer understanding of the changes associated with persistent ECM remodeling may improve our understanding of FPD pathogenesis and support the development of strategies to maintain footpad integrity and welfare in intensive poultry production.

Persistent ECM remodeling is a common feature of chronic tissue injury and contributes to altered tissue architecture and impaired repair (Deng et al., 2020; Wen et al., 2022). Sustained mechanical loading alters cell–matrix interactions and activates mechanotransduction-related pathways, resulting in prolonged fibroblast activation, excessive collagen deposition, and progressive tissue stiffening (Kittelsen et al., 2024; Liu et al., 2020; Long et al., 2022). Among the molecules involved in mechanosensing, integrin β1 is an important receptor that transmits extracellular mechanical cues to intracellular signaling pathways and has been associated with extracellular matrix remodeling and fibrotic responses (Kittelsen et al., 2024; Pang et al., 2023; Wen et al., 2022). However, whether mechanotransduction-related pathways are associated with persistent ECM remodeling during chronic FPD remains unclear.

In addition to host molecular changes, increasing evidence suggests that microbial and metabolic factors contribute to chronic tissue remodeling. Skin and gut microbiota play important roles in maintaining tissue homeostasis through interactions with host metabolism and immune responses, whereas microbial dysbiosis has been associated with impaired tissue repair and persistent inflammation (Belkaid & Segre, 2014; Byrd et al., 2018; Gallo & Nakatsuji, 2011; Tilg et al., 2020). These findings raise the possibility that microbial alterations and metabolic changes may interact with host during the progression of FPD. However, whether these biological processes are coordinated during persistent ECM remodeling remains unclear. In the present study, we combined histopathology with skin and gut microbiome profiling, transcriptomics, proteomics, and metabolomics to characterize the biological changes associated with chronic FPD. By integrating these datasets, we sought to investigate the host molecular, microbial, and metabolic alterations associated with persistent extracellular matrix remodeling during disease progression.

## Materials and methods

### Animal ethics statement

All animal procedures were conducted in accordance with the Guidelines for the Care and Use of Experimental Animals issued by the Ministry of Science and Technology of China. The experimental protocol was approved by the Animal Ethics Committee of the Hubei Academy of Agricultural Sciences (Approval No. 2024-620-000-001-015).

### Animal selection and sampling collection

Healthy ducks (Health) and ducks with naturally occurring footpad dermatitis (FPD) were selected from the same commercial flock maintained under identical housing and management conditions. FPD was diagnosed based on visible plantar lesions, whereas healthy ducks showed intact footpads without visible swelling, hyperkeratosis, or lesions. Ducks meeting these criteria were randomly selected for sample collection, with each duck considered an independent biological replicate.

Macroscopic images of footpads were recorded before sampling. Blood samples were collected from the wing vein, and serum was separated for metabolomic analysis. Footpad tissues were collected from the lesion sites of FPD ducks and the corresponding anatomical sites of healthy ducks for histological and multi-omics analyses. The number of biological replicates used for each analytical platform is specified separately in the corresponding sections below.

### Histological analysis

Footpad tissues (n = 3 per group) were fixed in 4% paraformaldehyde, embedded in paraffin, sectioned at 5 μm, and subjected to hematoxylin and eosin (H&E; Servicebio, G1005), Masson's trichrome (Servicebio, G1006), and Sirius Red staining (Servicebio, G1078) according to the manufacturer's instructions. H&E staining was used to evaluate tissue morphology, whereas Masson's trichrome and Sirius Red staining were performed to assess collagen deposition and extracellular matrix remodeling. Under polarized light, type I collagen appeared yellow–orange and type III collagen appeared green. Images were captured using a digital microscope (× 100), and six randomly selected fields from each section were analyzed for collagen-positive area and the collagen type I/III ratio.

### RNA sequencing and data analysis

Total RNA was extracted from duck footpad skin (n = 3 biological replicates per group) using TRIzol reagent (Thermo Fisher Scientific), and RNA integrity was evaluated using an Agilent 2100 Bioanalyzer. Only samples with RNA integrity numbers (RIN) > 7.0 were used for library construction. Sequencing libraries were prepared using a standard poly(A)-enriched RNA protocol and sequenced on an Illumina NovaSeq 6000 platform to generate 150-bp paired-end reads. Raw reads were quality-filtered using Cutadapt (v1.9) to remove reads containing adapters, poly(A) or poly(G), >5% unknown nucleotides (N), or >20% low-quality bases (Q ≤ 20). The quality of the resulting clean reads was further assessed using FastQC (v0.11.9) based on Q20, Q30, and GC content. Clean reads passing these quality-control procedures were used for subsequent transcriptomic analyses. Raw sequencing data have been deposited in the NCBI Sequence Read Archive (SRA) under accession number PRJNA1400782.

### Immunohistochemical staining

Paraffin-embedded footpad sections (5 μm) from three ducks per group (n = 3 biological replicates per group) were subjected to immunohistochemical staining using an anti-integrin β1 antibody (GB115173, Servicebio) according to the manufacturer's instructions. Immunoreactivity was visualized using 3,3′-diaminobenzidine (DAB) and counterstained with hematoxylin. Bright-field images were captured under identical imaging conditions, and staining intensity together with the positive area was quantified using ImageJ software. Six randomly selected fields were analyzed for each section, and the mean value of the six fields was calculated for each duck and used as one biological replicate for statistical analysis.

### Protein extraction, digestion, and LC-MS/MS analysis

Proteins were extracted from footpad tissues (approximately 25 mg; n = 4 biological replicates per group) using a urea-based lysis buffer containing protease inhibitors. Protein concentration was determined using the Bradford assay, and protein quality was assessed by SDS-PAGE before digestion. Equal amounts of protein (150 μg) were reduced, alkylated, digested with sequencing-grade trypsin at a protein-to-enzyme ratio of 50:1 for 14-16 h at 37°C. The resulting peptides were desalted using solid-phase extraction cartridges, and equal amounts of peptides from each sample were pooled and fractionated by high-pH reversed-phase chromatography prior to LC–MS/MS analysis. Peptide fractions were analyzed using a Thermo Vanquish Neo UHPLC system coupled to a Thermo Astral mass spectrometer operated in data-independent acquisition (DIA) mode. Protein identification and label-free quantification were performed using DIA-NN (v1.9.1) in DirectDIA (library-free) mode against the *Anas platyrhynchos* UniProt database. Trypsin/P was specified as the protease with up to two missed cleavages; carbamidomethylation of cysteine was set as a fixed modification, and methionine oxidation and protein N-terminal acetylation were set as variable modifications. Peptide-spectrum matches and proteins were filtered at an FDR < 1%, and only proteins passing these quality-control criteria were retained for subsequent quantitative and bioinformatic analyses. Differentially abundant proteins (DAPs) between the Health and FPD groups were identified using a two-sided Student's t-test, with *P* < 0.05 and fold change > 1.2 or < 0.83 as the significance thresholds.

### DNA extraction, 16S rRNA gene amplification, and sequencing

Microbial genomic DNA was extracted from footpad swabs (n = 8 biological replicates per group) and fecal samples (n = 6 biological replicates per group) using the Magnetic Soil and Stool DNA Kit (Tiangen Biotech, China). Each sample was obtained from an individual duck and represented one biological replicate. Extracted DNA was quantified using a Qubit fluorometer (Invitrogen, USA). The V3–V4 region of the bacterial 16S rRNA gene was amplified using universal primers 341F/805R (341F: CCTACGGGNGGCWGCAG; 805R: GACTACHVGGGTWTCTAATCC). Qualified libraries were sequenced on a DNBSEQ-G99 platform (MGI Tech, China) using paired-end sequencing. Library preparation and sequencing were performed according to the manufacturer's standard protocols.

Raw sequencing reads were processed using QIIME2 (v2025.7). After quality filtering, denoising, chimera removal, and amplicon sequence variant (ASV) inference using DADA2, taxonomy was assigned against the SILVA 138.2 database. Only quality-filtered, non-chimeric sequences were retained for downstream analyses. Alpha diversity indices (Chao1, ACE, Shannon, and Simpson) were calculated in QIIME2 and visualized in R. Beta diversity was assessed based on Bray–Curtis dissimilarity and visualized by principal coordinates analysis (PCoA), with group differences evaluated using permutational multivariate analysis of variance (PERMANOVA). Differential taxa were identified using the Wilcoxon rank-sum test and linear discriminant analysis effect size (LEfSe), with an LDA score > 3 used as the threshold. Functional profiles were predicted using PICRUSt2 based on the KEGG database.

### Non-target metabolome

Footpad tissues (approximately 50 mg; n = 6 biological replicates per group) and serum samples (100 μL; n = 6 biological replicates per group) were extracted with precooled 80% methanol and centrifuged to obtain metabolite-containing supernatants. After drying and reconstitution, metabolite profiling was performed using an UltiMate 3000 UPLC system (Thermo Fisher Scientific) coupled to a Q Exactive Orbitrap mass spectrometer (Thermo Fisher Scientific) operated in both positive and negative electrospray ionization modes. Chromatographic separation was achieved on an ACQUITY UPLC T3 column (Waters, USA). Pooled quality-control (QC) samples were prepared by combining equal aliquots (10 μL) of individual sample extracts and were analyzed after every 10 study samples to monitor LC–MS analytical stability.

Raw LC–MS data were converted into mzXML format and processed using XCMS, CAMERA, and metaX in R for peak detection, retention time alignment, feature annotation, and normalization. Metabolites were annotated against KEGG and HMDB databases and validated using an in-house MS/MS spectral library, with a mass tolerance of < 10 ppm used for database matching. Principal component analysis (PCA), hierarchical clustering, and partial least squares-discriminant analysis (PLS-DA) were used to evaluate metabolic variation among groups. Differential metabolites were identified based on Student's *t*-test (*P* < 0.05), fold change > 1.2, and variable importance in projection (VIP) > 1. KEGG pathway enrichment analysis was subsequently performed to identify significantly altered metabolic pathways.

### Statistical analyses

Statistical analyses were performed using GraphPad Prism 9.0 (GraphPad Software, USA). Data are presented as the mean ± standard error of the mean (SEM). Differences between the Health and FPD groups were evaluated using unpaired two-tailed Student's *t*-tests, and Pearson correlation analysis was used to assess associations among variables. For omics datasets, the statistical methods and thresholds used to identify differentially expressed genes, differentially abundant proteins, microbial taxa, and metabolites are specified in the corresponding Methods sections. Where multiple-testing correction was applied, the corresponding adjusted *P* values or false discovery rate (FDR) thresholds are specified for the relevant analysis. Effect sizes were reported as fold changes (or log₂ fold changes) for differential molecular features and as Pearson correlation coefficients (*r*) for correlation analyses. Statistical significance was defined as *P* < 0.05, whereas 0.05 ≤ *P* < 0.10 was considered a tendency toward significance.

## Results

### Chronic FPD is characterized by collagen disorganization and extracellular matrix remodeling

Gross and histopathological examination revealed marked structural alterations in FPD-affected footpads. Compared with healthy controls, FPD-affected footpads exhibited obvious thickening, deformation, and ulcerative lesions (Fig. 1A). Histological analysis further demonstrated pronounced hyperkeratosis, disruption of epidermal organization, loss of normal dermal architecture, and significantly higher pathological scores in FPD tissues (Fig. 1B, C).

**Fig. 1:**
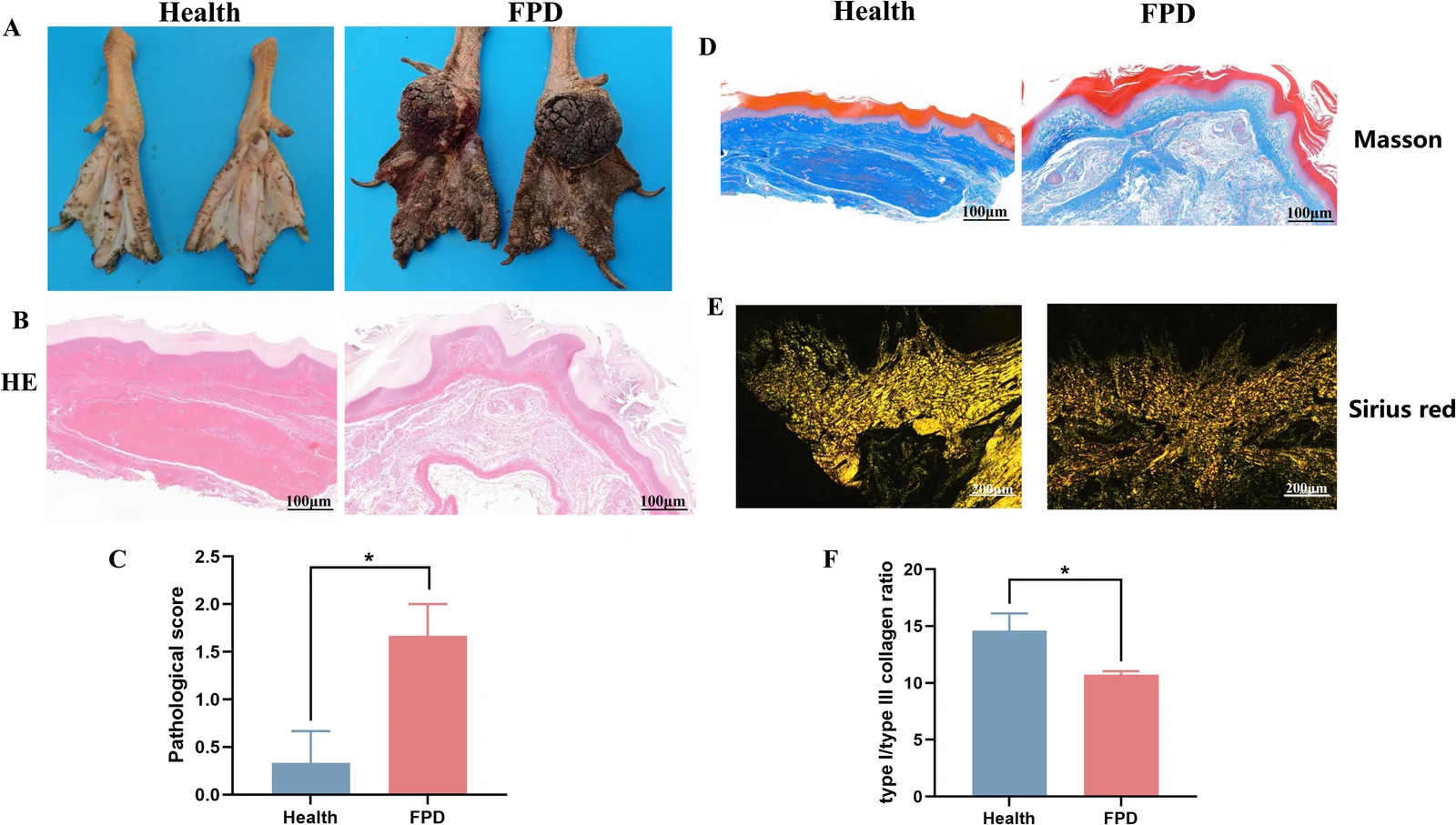
**Histopathological characterization of extracellular matrix (ECM) remodeling in duck footpads with footpad dermatitis (FPD).** (A) Representative gross morphology of healthy and FPD-affected footpads. (B) Representative hematoxylin and eosin (H&E) staining of footpad tissues. (C) Histopathological scores. (D) Representative Masson's trichrome staining of collagen fibers. (E) Representative Sirius Red staining under polarized light showing type I collagen (yellow/orange-red) and type III collagen (green). (F) Quantification of the collagen type I/type III ratio. Scale bars = 100 μm (B, D) and 200 μm (E). Health, healthy ducks; FPD, ducks with footpad dermatitis. n = 3 biological replicates per group. Data are presented as mean ± SEM. Differences between groups were analyzed using an unpaired two-tailed Student's *t*-test. *P* < 0.05 was considered statistically significant.

To further characterize extracellular matrix (ECM) alterations, collagen organization was examined using Masson's trichrome and Sirius Red staining. In healthy footpads, collagen fibers were dense and well organized, whereas FPD tissues exhibited fragmented, loosely arranged, and unevenly distributed collagen fibers (Fig. 1D). Sirius Red staining under polarized light further showed a significant reduction in the type I/type III collagen ratio in FPD tissues (Fig. 1E, F), indicating altered collagen composition. Collectively, these findings identify collagen disorganization and extracellular matrix remodeling as prominent pathological features of chronic FPD.

### Transcriptomic alterations associated with extracellular matrix remodeling

To investigate the molecular changes associated with extracellular matrix (ECM) remodeling, transcriptomic profiling was performed in healthy and FPD-affected footpads. Principal component analysis showed a clear separation between the two groups, with the first two principal components explaining 58.2% of the total variance (Fig. 2A). A total of 530 differentially expressed genes (DEGs) were identified, including 307 upregulated and 223 downregulated genes in FPD tissues (Figs. 2B, S1A). Functional enrichment analysis showed that these DEGs were mainly enriched in ECM organization, ECM–receptor interaction, focal adhesion, and TGF-β signaling (Fig. 2C).

**Fig. 2:**
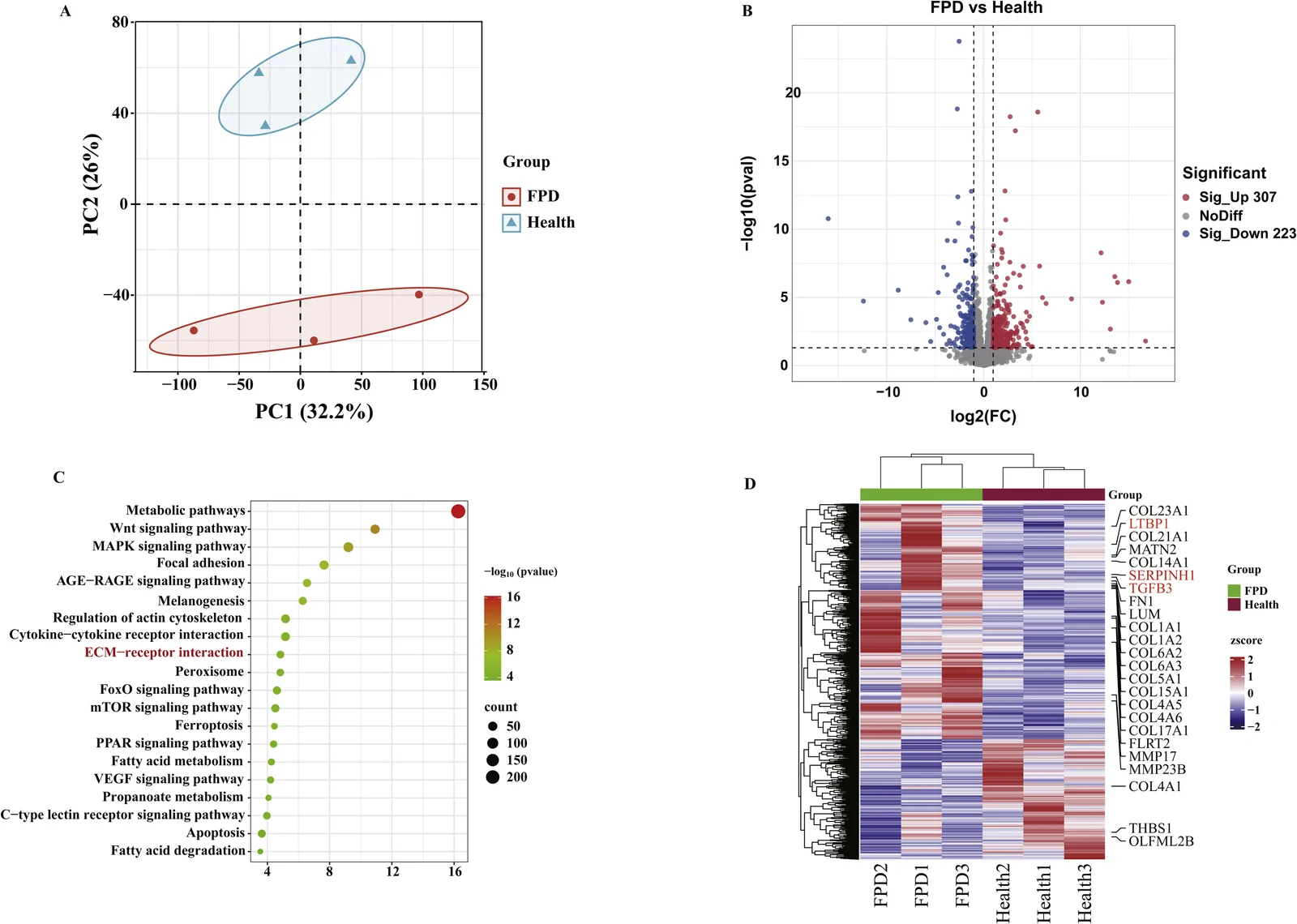
Transcriptomic alterations associated with extracellular matrix (ECM) remodeling in duck footpads with footpad dermatitis (FPD). (A) Principal component analysis (PCA) of transcriptomic profiles. (B) Volcano plot of differentially expressed genes (DEGs). (C) Kyoto Encyclopedia of Genes and Genomes (KEGG) pathway enrichment analysis of DEGs. DEGs were identified using *P* < 0.05 and |log₂ fold change| > 1. (D) Heatmap of representative DEGs involved in extracellular matrix organization, collagen formation, and cell adhesion. Health, healthy ducks; FPD, ducks with footpad dermatitis. n = 3 biological replicates per group.

Consistent with these enriched pathways, genes involved in collagen biosynthesis and extracellular matrix organization, including TGFB3, LTBP1, SERPINH1, COL1A1, COL1A2, COL6A2, COL6A3, COL15A1, FN1, LUM, and MATN2, were upregulated in FPD tissues, whereas COL4A1 and THBS1, which are associated with basement membrane organization and tissue homeostasis, were downregulated (Fig. 2D). ITGB1, a gene involved in cell–matrix adhesion and mechanotransduction, was also significantly upregulated (Figs. S1B, C). Weighted gene co-expression network analysis further identified FPD-associated gene modules enriched in ECM organization, TGF-β signaling, metabolism, and inflammatory regulation, whereas modules associated with healthy footpads were enriched in tissue maintenance and structural integrity (Figs. S1D-F). Collectively, these findings indicate that chronic FPD is accompanied by extensive transcriptional alterations associated with extracellular matrix remodeling and cell–matrix interactions.

### Proteomic alterations associated with extracellular matrix remodeling

To determine whether the transcriptional alterations were reflected at the protein level, quantitative proteomic analysis was performed. Principal component analysis clearly separated healthy and FPD samples, with PC1 and PC2 explaining 32.4% and 21.2% of the total variance, respectively (Fig. 3A). A total of 730 differentially abundant proteins (DAPs) were identified, including 121 increased and 609 decreased proteins in FPD tissues (Figs. 3B, S2A).

**Fig. 3:**
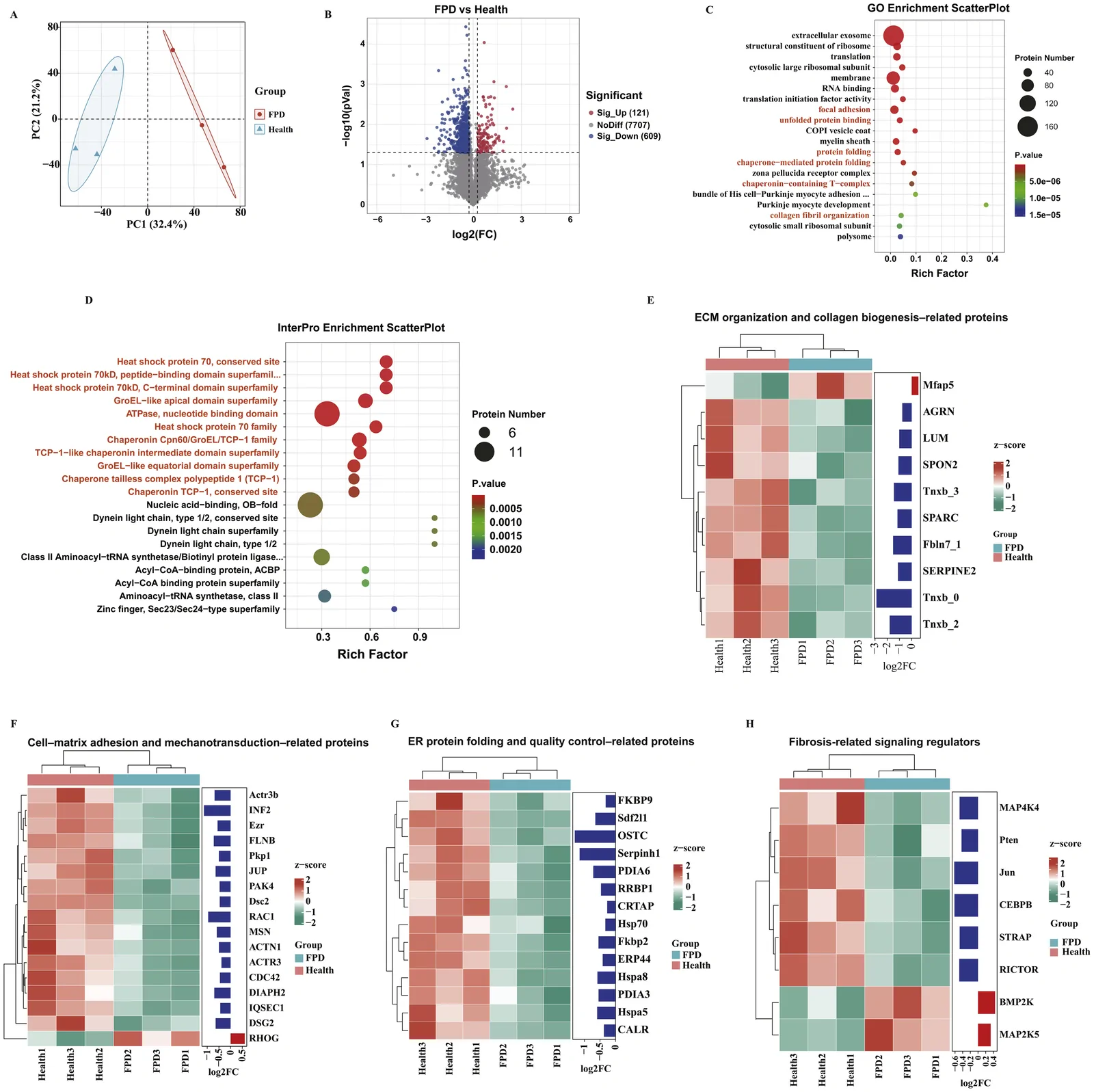
**Proteomic alterations associated with extracellular matrix organization in duck footpads with footpad dermatitis (FPD).** (A) Principal component analysis (PCA) of proteomic profiles. (B) Volcano plot of differentially abundant proteins (DAPs). DAPs were identified using *P* < 0.05 and fold change > 1.2 or < 0.83. (C) Gene Ontology (GO) enrichment analysis of DAPs. (D) InterPro domain enrichment analysis. (E–G) Heatmaps of representative DAPs involved in (E) extracellular matrix organization, (F) cell–matrix adhesion and mechanotransduction, and (G) protein folding and protein quality control. (H) Representative proteins involved in TGF-β signaling, integrin-mediated mechanotransduction, and extracellular matrix organization. Health, healthy ducks; FPD, ducks with footpad dermatitis. n = 4 biological replicates per group.

Functional enrichment analysis showed that the DAPs were mainly associated with collagen fibril organization, ECM–receptor interaction, focal adhesion, protein folding, and chaperone-mediated processes (Fig. 3C; Fig. S2B). Domain enrichment further identified heat shock proteins, chaperonins, and ATPase-related proteins, including members of the HSP70, HSP90, and TRiC complexes (Fig. 3D). Consistent with these enrichment results, multiple proteins involved in extracellular matrix organization, collagen remodeling, and cell–matrix adhesion were differentially abundant in FPD tissues. Proteins associated with TGF-β-related signaling, including MFAP5, RICTOR, BMP2K, and MAP2K5, were also markedly altered (Fig. 3E-H). Collectively, these findings indicate that chronic FPD is accompanied by extensive proteomic alterations associated with extracellular matrix organization, cell–matrix interactions, and protein homeostasis.

### Microbial alterations associated with extracellular matrix remodeling

Because persistent ECM remodeling is closely linked to the local tissue microenvironment, the footpad microbiota was further characterized. Neither Chao1 richness nor Shannon diversity differed significantly between healthy and FPD groups (Fig. 4A, B). PLS-DA showed visual separation between the two groups; however, PCoA based on Bray–Curtis distances did not reveal a significant difference in overall community composition (Figs. 4C, S3A-C). Taxonomic analysis showed enrichment of Fusobacteriota, Actinomycetota, and Streptococcus in FPD-affected footpads, together with depletion of Ligilactobacillus (Figs. 4D-F, S3D). In contrast, healthy footpads exhibited higher abundances of Lactobacillus salivarius, Jeotgalicoccus, and Brachybacterium. Functional prediction identified reduced microbial functions related to amino acid and lipid metabolism together with increased functions associated with translation and replication (Fig. S3E), indicating functional alterations within the local microbial community.

**Fig. 4:**
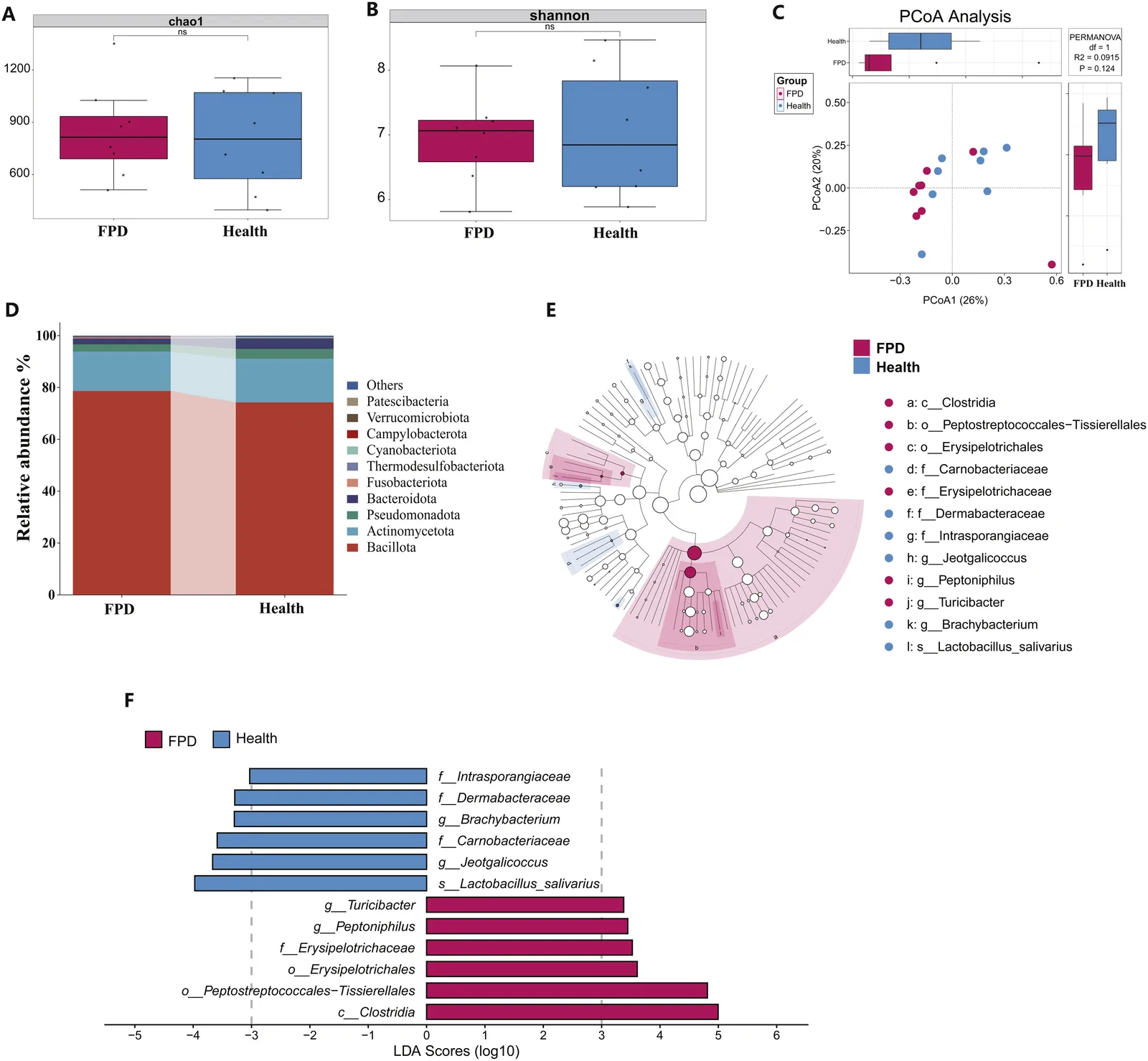
**Microbial community alterations in duck footpads with footpad dermatitis (FPD).** (A-B) Alpha diversity of the footpad microbiota based on the Chao1 richness and Shannon diversity indices. (C) Principal coordinates analysis (PCoA) based on Bray–Curtis distances. Group differences in microbial community composition were evaluated using permutational multivariate analysis of variance (PERMANOVA). (D) Relative abundance of the dominant bacterial phyla. (E) Linear discriminant analysis effect size (LEfSe) cladogram of differentially abundant bacterial taxa. (F) Linear discriminant analysis (LDA) scores of differentially abundant bacterial taxa identified by LEfSe (LDA score > 3). Health, healthy ducks; FPD, ducks with footpad dermatitis. n = 8 biological replicates per group.

The gut microbiota was subsequently analyzed to determine whether similar alterations were present beyond the footpad lesion. Similar compositional changes were observed in the intestine (Figs. S4A-C), with enrichment of Fusobacteriota, Streptococcus, Megamonas, and Rikenellaceae_RC9_gut_group in FPD ducks, whereas healthy ducks showed higher abundances of Bacteroidota and Romboutsia (Figs. S4D, E). LEfSe analysis further identified several Fusobacterium-related taxa as characteristic features of the FPD-associated gut microbiota (LDA > 3; Fig. S4F). Collectively, these findings indicate that chronic FPD is associated with alterations in both local and gut microbial communities.

### Metabolic alterations associated with extracellular matrix remodeling

Given the predicted alterations in microbial metabolic functions, metabolomic profiling was performed to further characterize metabolic changes associated with ECM remodeling. Untargeted LC–MS metabolomics clearly separated healthy and FPD footpad samples in principal component analysis (Fig. 5A). A total of 595 differential metabolites were identified, including 448 increased and 147 decreased metabolites in FPD tissues (Fig. 5B). Hierarchical clustering further distinguished the metabolic profiles of healthy and FPD footpads (Fig. 5C). Functional enrichment analysis showed that these metabolites were mainly involved in nucleotide metabolism, purine metabolism, cofactor biosynthesis, and several signaling-related pathways (Fig. 5D). Representative metabolites, including niacinamide, uridine 5′-monophosphate, and guanosine, were increased in FPD tissues, whereas cortisol was decreased.

**Fig. 5:**
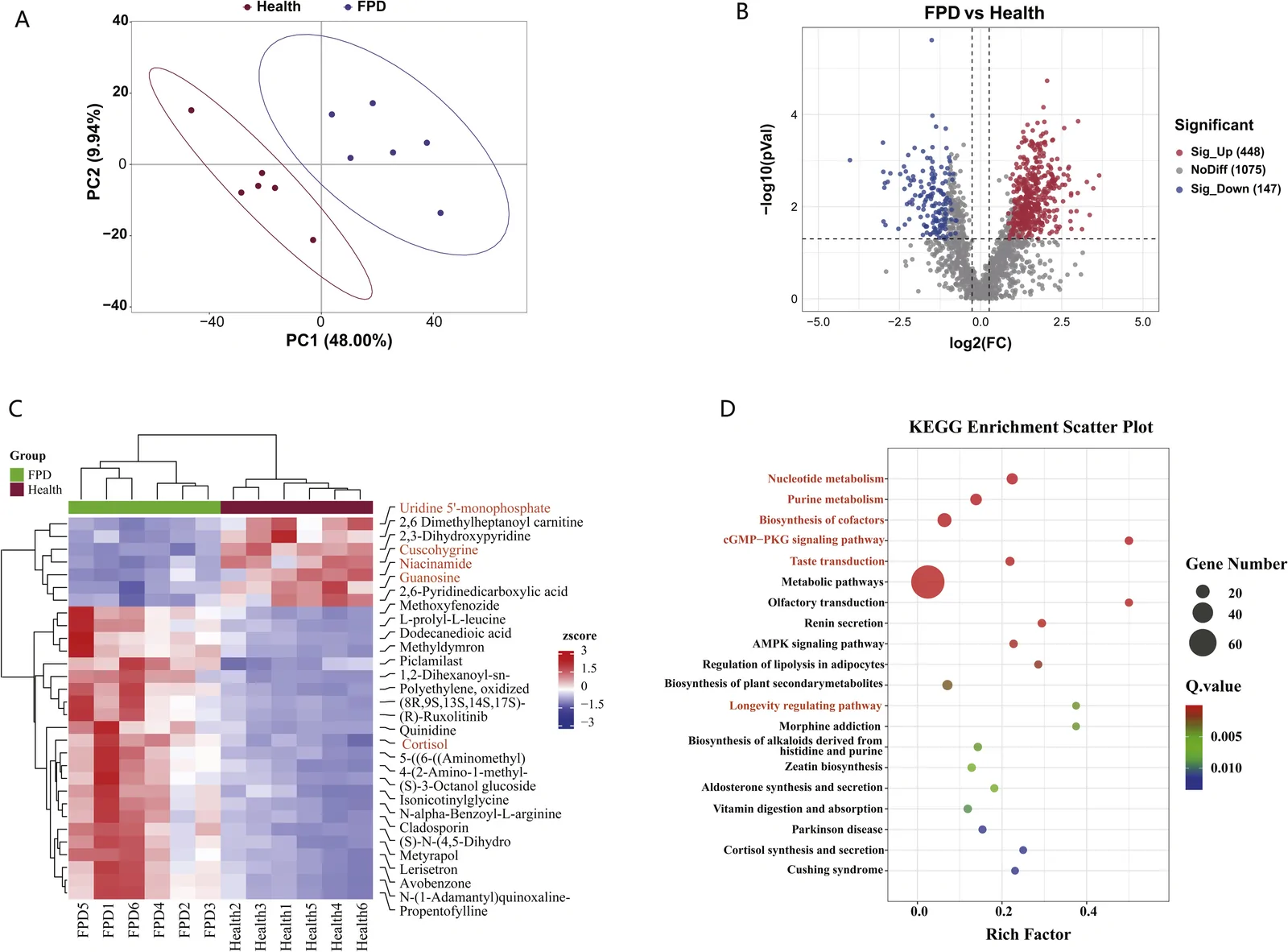
**Metabolic alterations in duck footpads with footpad dermatitis (FPD).** (A) Principal component analysis (PCA) of metabolomic profiles. (B) Volcano plot of differential metabolites. Differential metabolites were identified using *P* < 0.05, fold change > 1.2, and variable importance in projection (VIP) > 1. (C) Hierarchical clustering of representative differential metabolites. (D) Kyoto Encyclopedia of Genes and Genomes (KEGG) pathway enrichment analysis of differential metabolites. Health, healthy ducks; FPD, ducks with footpad dermatitis. n = 6 biological replicates per group.

Serum metabolomic profiling was subsequently performed to determine whether similar metabolic alterations were present beyond the footpad lesion. Principal component analysis also separated healthy and FPD serum samples (Fig. S5A), and 34 differential metabolites associated with amino acid, lipid, and energy metabolism were identified (Figs. S5B, C). Enrichment analysis highlighted oxidative phosphorylation, purine metabolism, sulfur metabolism, and ferroptosis-related pathways (Fig. S5D). Several metabolites were detected in both footpad tissue and serum. Collectively, these findings indicate that chronic FPD is associated with metabolic alterations in both local tissues and the systemic circulation.

### Integrated multi-omics associations related to extracellular matrix remodeling

To examine the relationships among microbial, metabolic, and host molecular alterations, integrated multi-omics correlation analysis was performed. Strong correlations were observed among microbial composition, differential metabolites, and host molecular features associated with extracellular matrix remodeling. Several FPD-associated microbial genera were significantly correlated with differential metabolites, including cuscohygrine, guanosine, uridine 5′-monophosphate, cortisol, and niacinamide (Fig. S6A).

Integration of metabolomic and transcriptomic datasets showed that these metabolites were significantly correlated with ECM-related genes, including TGFB3, LTBP1, and SERPINH1 (Fig. 6A). Similar correlations were observed at the protein level, where guanosine and niacinamide were positively correlated with MFAP5, and MFAP5 was correlated with structural ECM proteins, including COL17A1 and THBS1 (Figs. 6B, S6B). Collectively, these results demonstrate correlations among microbial composition, metabolites, and host molecular features associated with extracellular matrix remodeling in chronic FPD.

**Fig. 6:**
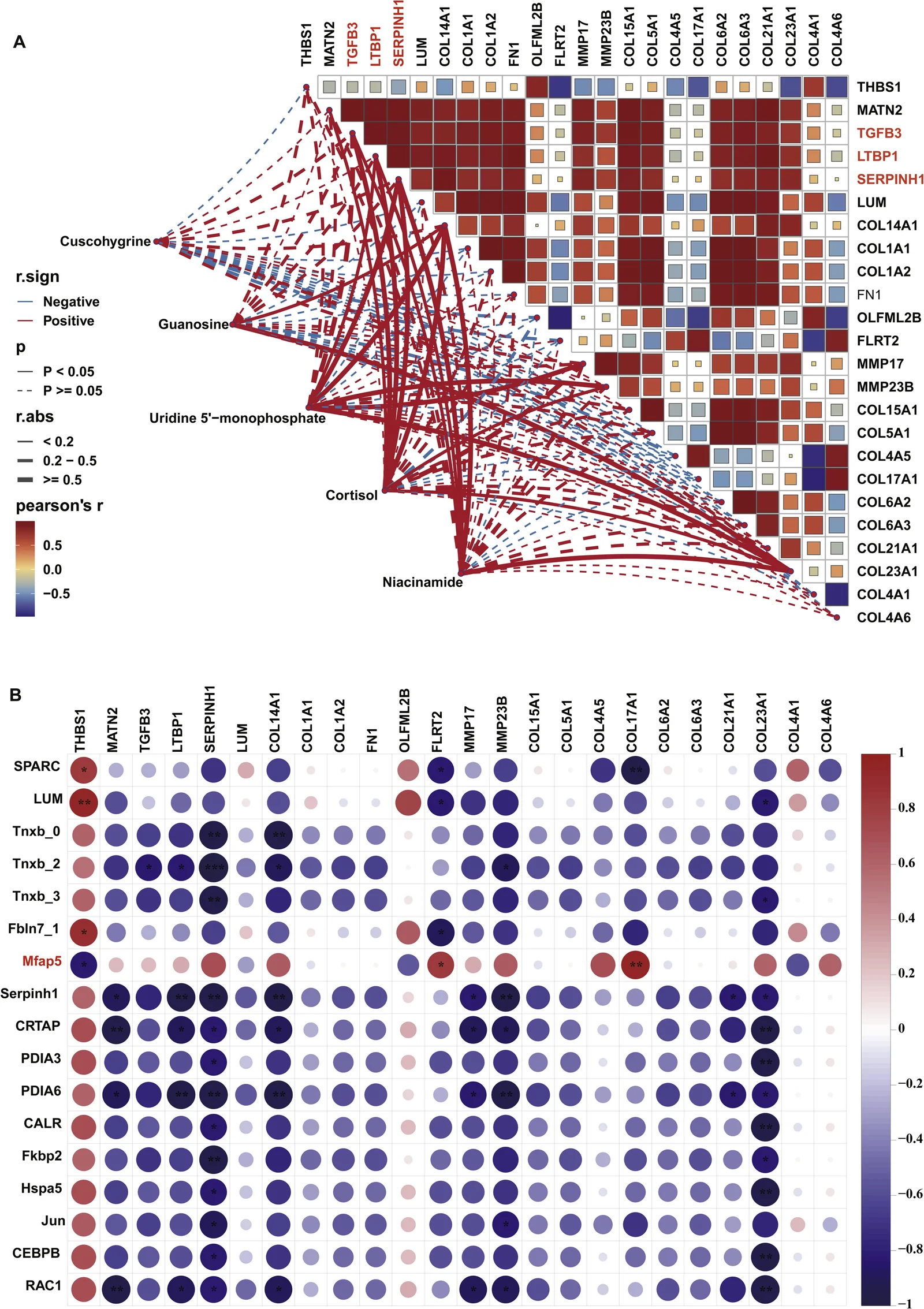
**Integrated multi-omics correlation analysis of host molecular, microbial, and metabolic alterations.** (A) Correlation network of representative microbial taxa, differential metabolites, and extracellular matrix (ECM)-related genes. Nodes represent microbial taxa, metabolites, or genes, and edges represent correlations between variables. Red and blue lines indicate positive and negative correlations, respectively. (B) Correlation network of representative ECM-related genes and differentially abundant proteins involved in extracellular matrix remodeling, mechanotransduction, and protein homeostasis. Correlations were assessed using Pearson's correlation analysis. The correlations shown represent statistical associations and do not imply causal relationships.

## Discussion

Footpad dermatitis (FPD) is generally considered a chronic inflammatory condition resulting from prolonged exposure to unfavorable flooring conditions. In the present study, histological and multi-omics analyses identified persistent extracellular matrix (ECM) remodeling as a characteristic feature of chronic FPD. FPD lesions were characterized by epidermal disruption, hyperkeratosis, disorganized collagen architecture, and a marked reduction in the type I/type III collagen ratio. During normal wound healing, type III collagen is deposited during the early repair phase and is gradually replaced by mechanically stronger type I collagen as tissue maturation progresses (Singh et al., 2023). Therefore, the persistently reduced type I/type III collagen ratio observed in FPD lesions is consistent with impaired matrix maturation and continued collagen remodeling. These findings suggest that structural remodeling persists in established FPD lesions and that normal tissue architecture is not fully restored. These findings highlight persistent extracellular matrix remodeling as an important pathological feature of chronic FPD in addition to chronic inflammation.

Transcriptomic and proteomic analyses further showed extensive host molecular alterations associated with persistent ECM remodeling in FPD. Differentially expressed genes and proteins were consistently enriched in ECM–receptor interaction, focal adhesion, TGF-β signaling, collagen organization, extracellular matrix assembly, and cell adhesion. These pathways have established roles in fibroblast activation, collagen deposition, and extracellular matrix turnover during chronic tissue remodeling (Henderson & Sheppard, 2013; Meng et al., 2016), supporting their potential involvement in the structural alterations observed in FPD. Among the altered molecules, ITGB1 was markedly upregulated. Integrin β1 is a key mediator linking extracellular mechanical stimuli to intracellular signaling and has been implicated in fibroblast activation and extracellular matrix remodeling under persistent mechanical stress (Reed et al., 2015). Recent poultry studies have further shown that housing and flooring conditions are associated with footpad health, highlighting the potential relevance of the physical environment to FPD (Biesek et al., 2024). Given the continuous weight-bearing and friction experienced by the duck footpad, the observed changes in integrin-associated signaling may reflect a mechanotransduction response accompanying persistent ECM remodeling (Saraswathibhatla et al., 2023). However, mechanical loading was not experimentally manipulated in the present study, and the functional role of integrin β1/TGF-β signaling in ECM remodeling remains to be established. Proteomic analysis also identified enrichment of protein folding and chaperone-related processes together with increased abundance of heat shock proteins. Molecular chaperones are required for collagen folding, extracellular matrix assembly, and protein quality control during tissue repair (Kanchanawong & Calderwood, 2023). These findings are consistent with increased protein quality control during extracellular matrix remodeling. Although transcriptomic and proteomic changes were not always consistent at the individual gene or protein level, both datasets identified similar biological pathways related to extracellular matrix organization, cell–matrix interactions, and protein homeostasis.

In addition to host molecular alterations, chronic FPD was also associated with changes in the local microbiota and metabolic profile. The microbial community was characterized by selective shifts in composition rather than an overall loss of diversity, including enrichment of *Streptococcus, Fusobacterium*, and *Actinomycetota*, together with depletion of potentially beneficial taxa such as *Ligilactobacillus*. Similar microbial changes have been reported in chronic barrier-associated disorders, where disease progression is associated with redistribution of specific microbial taxa rather than widespread disruption of the microbial community (Belkaid & Harrison, 2017; Gopalakrishnan et al., 2018; Sinha et al., 2021). These findings indicate that chronic FPD is associated with a distinct local microbial community. Metabolomic analysis further identified alterations in pathways related to amino acid, lipid, nucleotide, and redox metabolism (Franzosa et al., 2019; Jin et al., 2024; Wynn, 2008). Changes in metabolites involved in nucleotide metabolism, including guanosine and uridine 5′-monophosphate, together with altered niacinamide and cortisol levels, further indicate changes in local metabolic processes. Similar microbial alterations in the gut together with metabolic changes in serum suggest that these alterations are not limited to the footpad lesion but are also present beyond the local lesion.

Integrated multi-omics analysis identified associations among host molecular alterations, microbial composition, and metabolic profiles in chronic FPD. Correlation analysis further showed significant relationships among representative microbial taxa, differential metabolites, and ECM-related genes and proteins (Koh et al., 2016). Together with the enrichment of mechanotransduction-related pathways, these findings indicate that persistent ECM remodeling in FPD occurs alongside changes in host mechanotransduction-related signaling and the microbial and metabolic microenvironment. Similar alterations in the gut microbiota and serum metabolome further suggest that the biological changes associated with FPD are not restricted to the local footpad lesion (Dai et al., 2025; Wu et al., 2017). Importantly, the cross-omics relationships identified in the present study are correlative and do not establish causal relationships among microbial alterations, metabolic changes, host molecular responses, and ECM remodeling. Likewise, although the enrichment of mechanotransduction-related pathways is consistent with a possible contribution of mechanical loading to chronic FPD, this relationship was not directly tested in the present study. A further limitation is the lack of functional experiments directly testing the role of integrin β1/TGF-β signaling in ECM remodeling. Although histological, transcriptomic, proteomic, and immunohistochemical findings consistently showed alterations in ECM organization and mechanotransduction-related signaling, these observations do not establish a functional role for this pathway in chronic FPD. Future studies combining controlled mechanical stimulation with targeted inhibition or knockdown of integrin β1/TGF-β signaling in primary duck footpad fibroblasts will be needed to determine whether this pathway directly contributes to fibroblast activation and ECM remodeling.

## Conclusion

This study shows that histopathological, transcriptomic, and proteomic evidence consistently supports persistent extracellular matrix (ECM) remodeling as a prominent pathological feature of chronic footpad dermatitis (FPD). Chronic FPD was further accompanied by extensive alterations in host molecular responses, microbial composition, and metabolic profiles. By integrating histopathology with transcriptomic, proteomic, microbiome, and metabolomic analyses, this study provides an integrated view of the biological changes associated with chronic FPD. Although the present study identifies associations rather than causal relationships among host molecular responses, microbial alterations, and metabolic changes, the functional role of integrin β1/TGF-β-related signaling in ECM remodeling remains to be experimentally validated. These findings extend the current understanding of FPD beyond chronic inflammation and provide a foundation for future mechanistic studies to clarify the processes underlying persistent ECM remodeling and to inform the development of nutritional, breeding, and management strategies for improving footpad health, animal welfare, and production sustainability in intensive poultry production.

## Author contributions

Cui Ma conceived the study and designed the experiments. Cui Ma, Yunhe Zong, Hao Zhang, Zhenhua Liang, and Ailuan Pan contributed to sample collection, experimental procedures, data acquisition, analysis, and interpretation. Jinsong Pi and Yan Wu contributed to the study design, interpretation of the results, supervision of the research, and acquisition of funding. Cui Ma drafted the manuscript. All authors critically reviewed and revised the manuscript for important intellectual content, approved the exact final version of the manuscript, and agree to be personally and publicly accountable for all aspects of the work, including ensuring that any questions related to the accuracy or integrity of the work are appropriately investigated and resolved.

## Disclosures

The authors declare that they have no conflicts of interest.

## Footnotes

Supplementary material associated with this article can be found, in the online version, at doi:10.1016/j.psj.2026.107830.
