# Supplementary material for: Integrated multi-omics reveals persistent extracellular matrix remodeling in chronic duck footpad dermatitis
Source: Poult Sci. 2026 Sep 23;105(12):107830. doi: 10.1016/j.psj.2026.107830 (PMC13636073; doi:10.1016/j.psj.2026.107830)
Supplement: Supplementary file 1 [file mmc1.pdf]

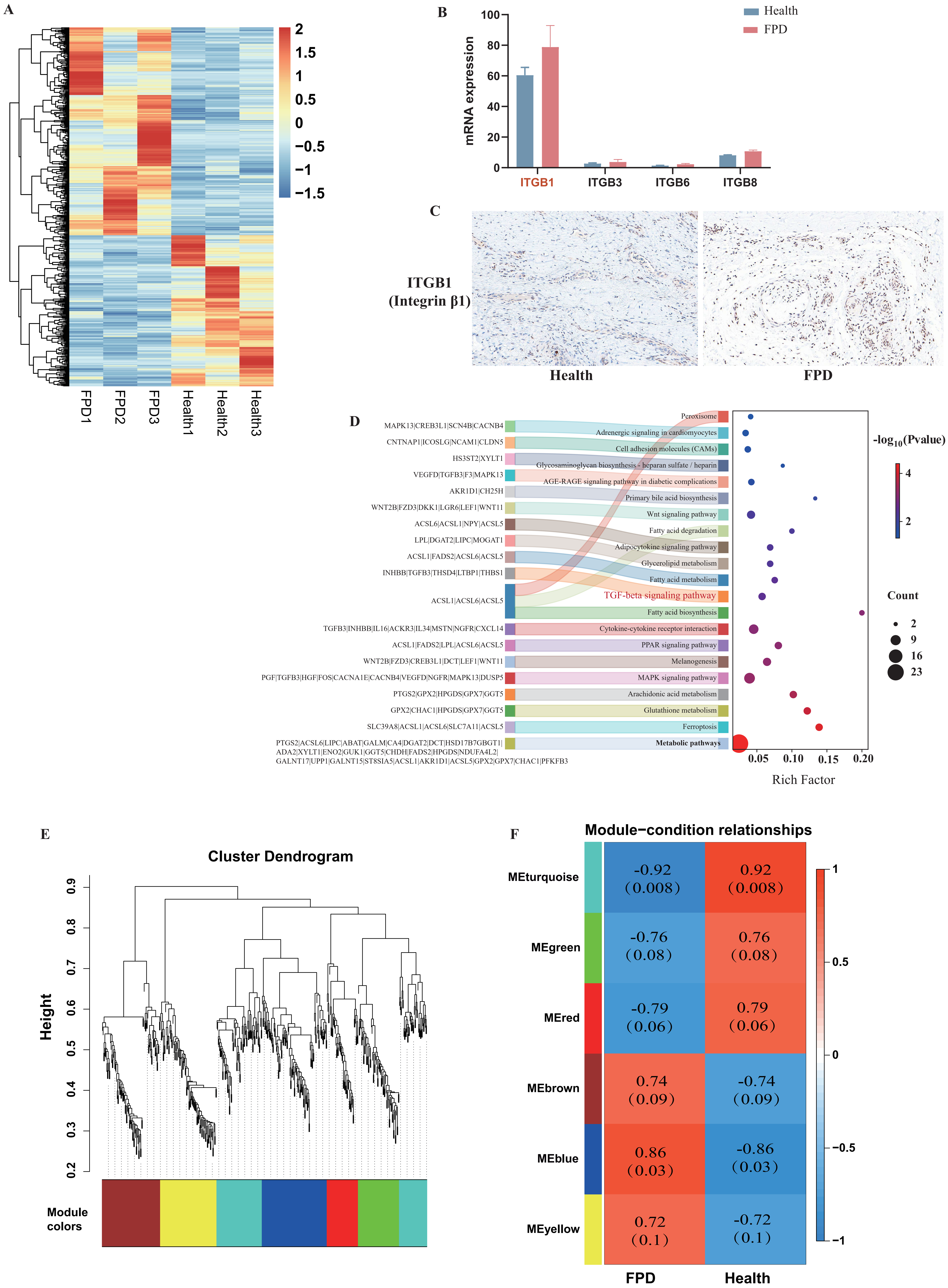

A
2
1.5
1
0.5
0
−0.5
−1
−1.5
FPD1
FPD2
FPD3
Health1
Health2
Health3
B
Health
FPD
mRNA expression
100
80
60
40
20
0
ITGB1
ITGB3
ITGB6
ITGB8
C
ITGB1
(Integrin β1)
Health
FPD
D
MAPK13|CREB3L1|SCN4B|CACNB4
CNTNAP1|ICOSLG|NCAM1|CLDN5
HS3ST2|XYLT1
VEGFD|TGFB3|F3|MAPK13
AKR1D1|CH25H
WNT2B|FZD3|DKK1|LGR6|LEF1|WNT11
ACSL6|ACSL1|NPY|ACSL5
LPL|DGAT2|LIPC|MOGAT1
ACSL1|FADS2|ACSL6|ACSL5
INHBB|TGFB3|THSD4|LTBP1|THBS1
ACSL1|ACSL6|ACSL5
TGFB3|INHBB|IL16|ACKR3|IL34|MSTN|NGFR|CXCL14
ACSL1|FADS2|LPL|ACSL6|ACSL5
WNT2B|FZD3|CREB3L1|DCT|LEF1|WNT11
PGF|TGFB3|HGF|FOS|CACNA1E|CACNB4|VEGFD|NGFR|MAPK13|DUSP5
PTGS2|GPX2|HPGDS|GPX7|GGT5
GPX2|CHAC1|HPGDS|GPX7|GGT5
SLC39A8|ACSL1|ACSL6|SLC7A11|ACSL5
PTGS2|ACSL6|LIPC|ABAT|GALM|CA4|DGAT2|DCT|HSD17B7GBGT1|
ADA2|XYLT1|ENO2|GUK1|GGT5|CHDH|FADS2|HPGDS|NDUFA4L2|
GALNT17|UPP1|GALNT15|ST8SIA5|ACSL1|AKR1D1|ACSL5|GPX2|GPX7|CHAC1|PFKFB3
Peroxisome
Adrenergic signaling in cardiomyocytes
Cell adhesion molecules (CAMs)
Glycosaminoglycan biosynthesis - heparan sulfate / heparin
AGE-RAGE signaling pathway in diabetic complications
Primary bile acid biosynthesis
Wnt signaling pathway
Fatty acid degradation
Adipocytokine signaling pathway
Glycerolipid metabolism
Fatty acid metabolism
TGF-beta signaling pathway
Fatty acid biosynthesis
Cytokine-cytokine receptor interaction
PPAR signaling pathway
Melanogenesis
MAPK signaling pathway
Arachidonic acid metabolism
Glutathione metabolism
Ferroptosis
Metabolic pathways
0.05
0.10
0.15
0.20
Rich Factor
-log10(Pvalue)
4
2
Count
2
9
16
23
E
Cluster Dendrogram
Height
0.9
0.8
0.7
0.6
0.5
0.4
0.3
0.2
Module colors
F
Module−condition relationships
MEturquoise
-0.92 (0.008)
0.92 (0.008)
MEgreen
-0.76 (0.08)
0.76 (0.08)
MEred
-0.79 (0.06)
0.79 (0.06)
MEbrown
0.74 (0.09)
-0.74 (0.09)
MEblue
0.86 (0.03)
-0.86 (0.03)
MEyellow
0.72 (0.1)
-0.72 (0.1)
FPD
Health
1
0.5
0
−0.5
−1
