# Supplementary material for: Integrated multi-omics reveals persistent extracellular matrix remodeling in chronic duck footpad dermatitis
Source: Poult Sci. 2026 Sep 23;105(12):107830. doi: 10.1016/j.psj.2026.107830 (PMC13636073; doi:10.1016/j.psj.2026.107830)
Supplement: Supplementary file 2 [file mmc2.pdf]

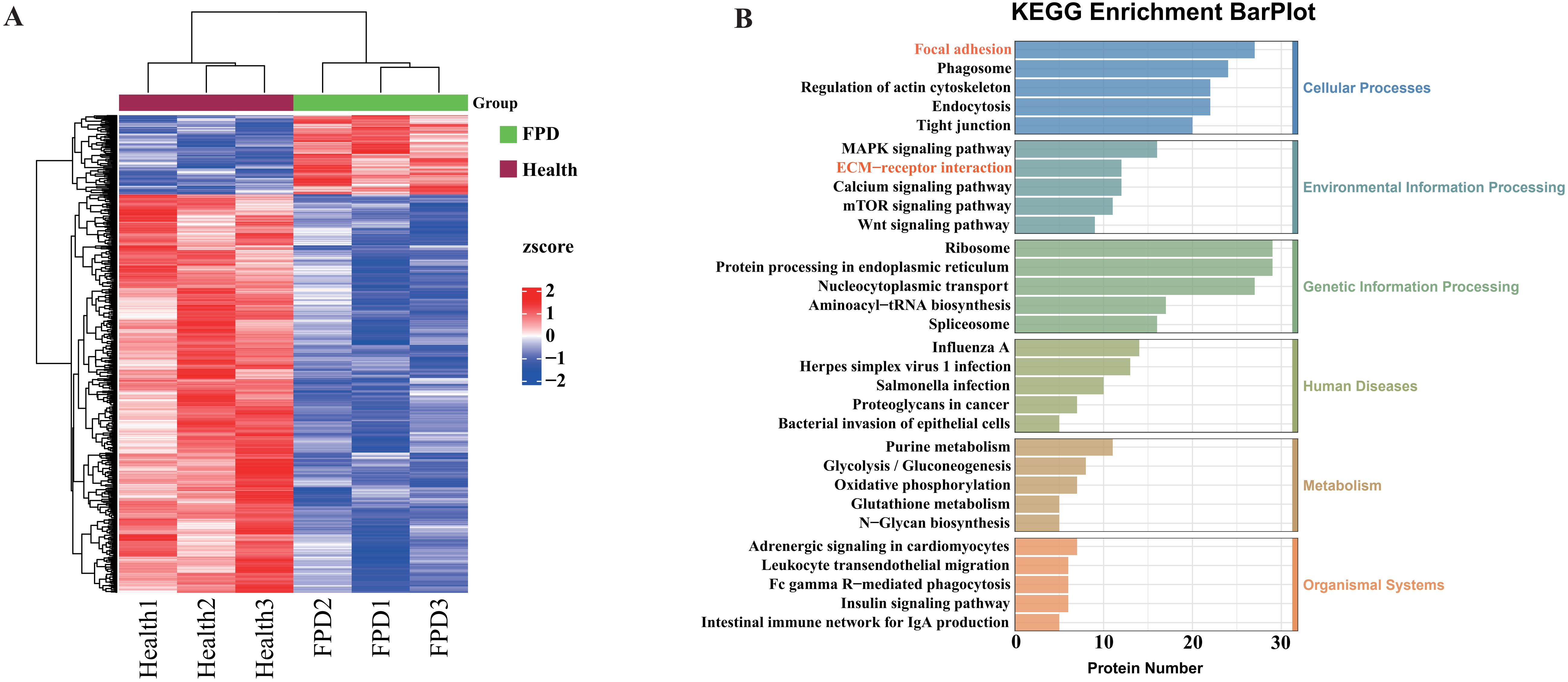
A
Group
FPD
Health
zscore
2
1
0
−1
−2
Health1
Health2
Health3
FPD2
FPD1
FPD3
B
KEGG Enrichment BarPlot
Focal adhesion
Phagosome
Regulation of actin cytoskeleton
Endocytosis
Tight junction
MAPK signaling pathway
ECM−receptor interaction
Calcium signaling pathway
mTOR signaling pathway
Wnt signaling pathway
Ribosome
Protein processing in endoplasmic reticulum
Nucleocytoplasmic transport
Aminoacyl−tRNA biosynthesis
Spliceosome
Influenza A
Herpes simplex virus 1 infection
Salmonella infection
Proteoglycans in cancer
Bacterial invasion of epithelial cells
Purine metabolism
Glycolysis / Gluconeogenesis
Oxidative phosphorylation
Glutathione metabolism
N−Glycan biosynthesis
Adrenergic signaling in cardiomyocytes
Leukocyte transendothelial migration
Fc gamma R−mediated phagocytosis
Insulin signaling pathway
Intestinal immune network for IgA production
Cellular Processes
Environmental Information Processing
Genetic Information Processing
Human Diseases
Metabolism
Organismal Systems
0
10
20
30
Protein Number
