# Supplementary material for: Integrated multi-omics reveals persistent extracellular matrix remodeling in chronic duck footpad dermatitis
Source: Poult Sci. 2026 Sep 23;105(12):107830. doi: 10.1016/j.psj.2026.107830 (PMC13636073; doi:10.1016/j.psj.2026.107830)
Supplement: Supplementary file 3 [file mmc3.pdf]

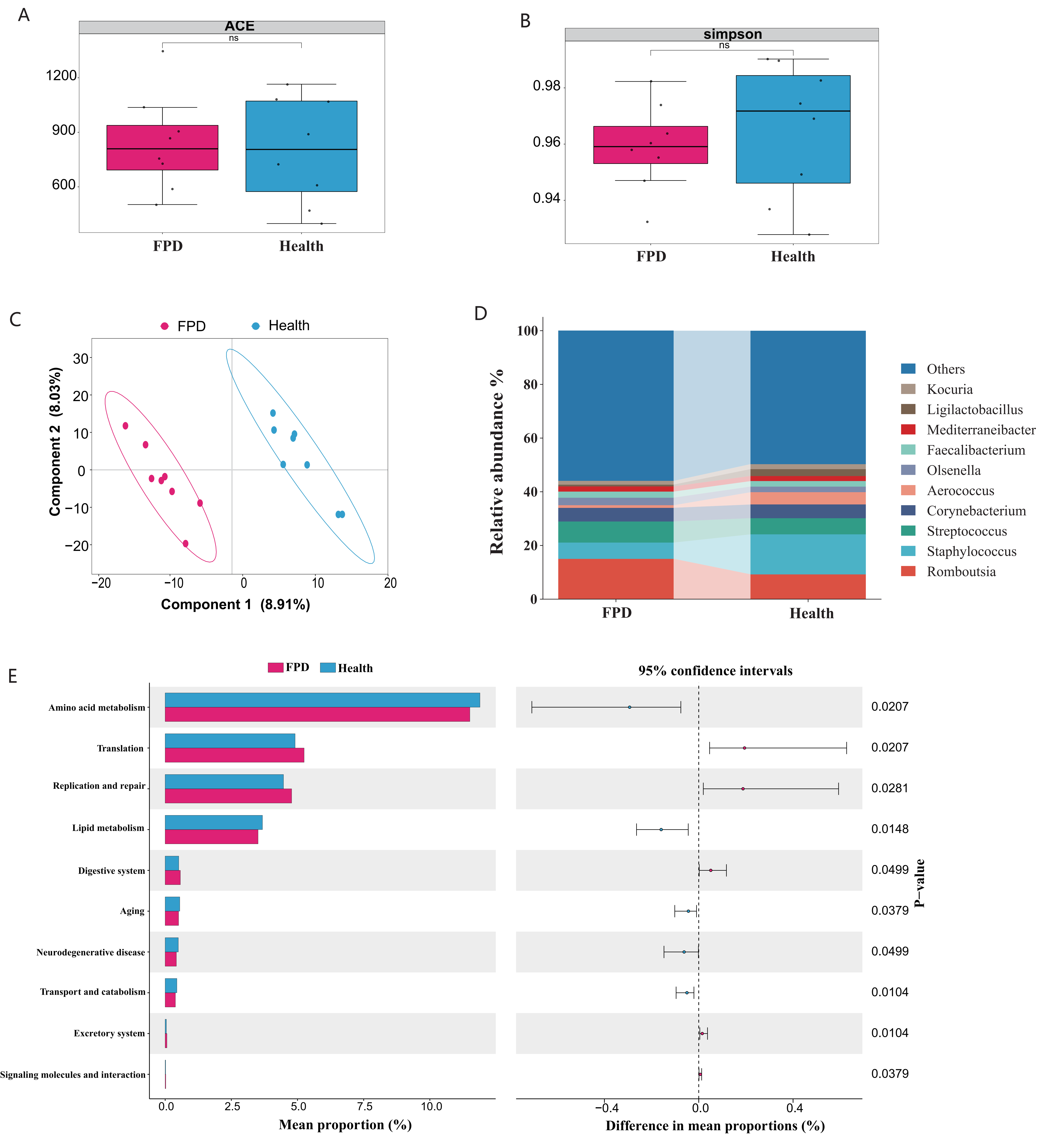

A
ACE
ns
1200
900
600
FPD
Health
B
simpson
ns
0.98
0.96
0.94
FPD
Health
C
FPD
Health
Component 2 (8.03%)
Component 1 (8.91%)
D
Relative abundance %
FPD
Health
Others
Kocuria
Ligilactobacillus
Mediterraneibacter
Faecalibacterium
Olsenella
Aerococcus
Corynebacterium
Streptococcus
Staphylococcus
Romboutsia
E
FPD
Health
95% confidence intervals
Amino acid metabolism
Translation
Replication and repair
Lipid metabolism
Digestive system
Aging
Neurodegenerative disease
Transport and catabolism
Excretory system
Signaling molecules and interaction
0.0207
0.0207
0.0281
0.0148
0.0499
0.0379
0.0499
0.0104
0.0104
0.0379
P−value
Mean proportion (%)
Difference in mean proportions (%)
