# Supplementary material for: Integrated multi-omics reveals persistent extracellular matrix remodeling in chronic duck footpad dermatitis
Source: Poult Sci. 2026 Sep 23;105(12):107830. doi: 10.1016/j.psj.2026.107830 (PMC13636073; doi:10.1016/j.psj.2026.107830)
Supplement: Supplementary file 4 [file mmc4.pdf]

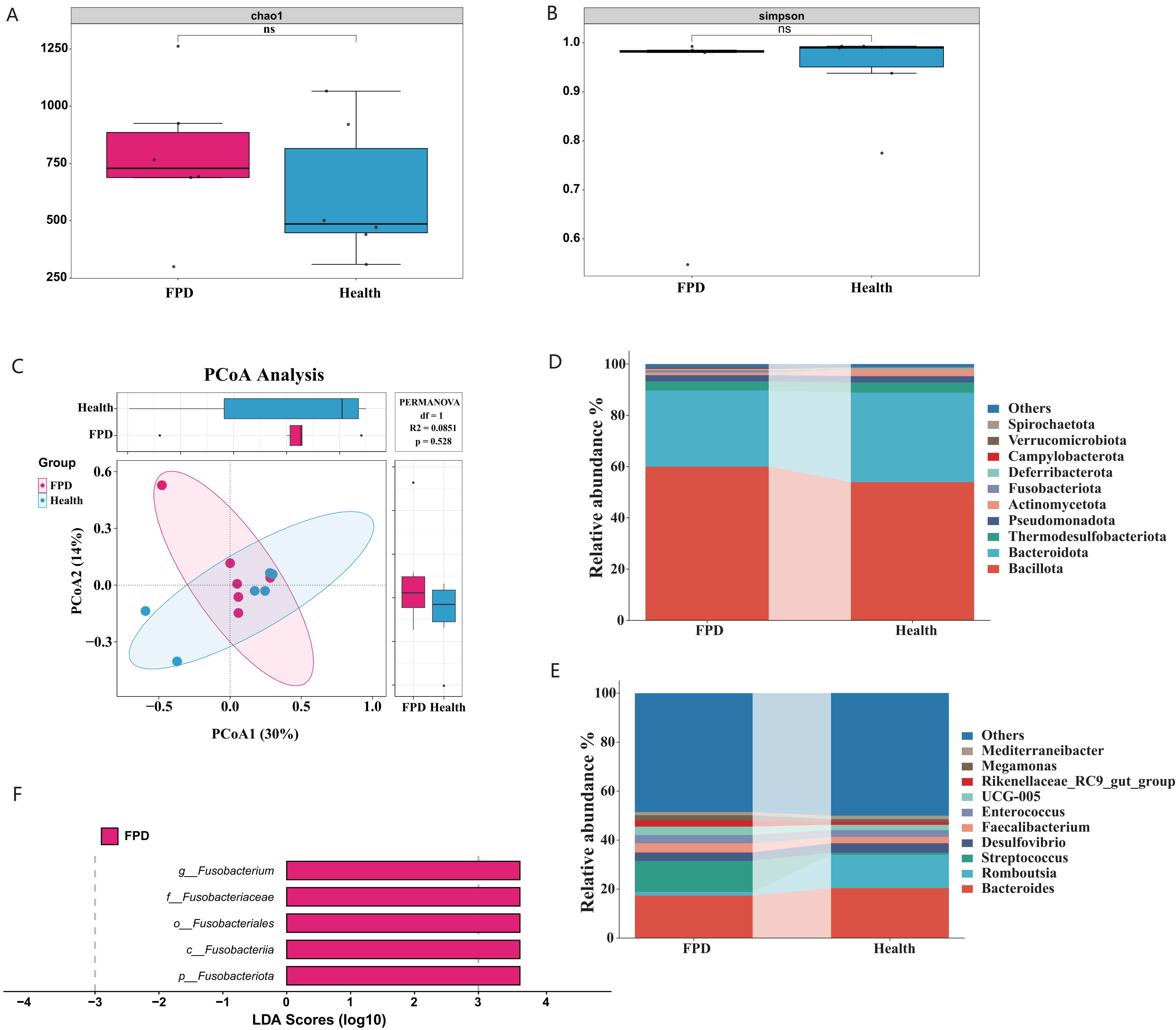

A
chao1
ns
1250
1000
750
500
250
FPD
Health
B
simpson
ns
1.0
0.9
0.8
0.7
0.6
FPD
Health
C
PCoA Analysis
Health
FPD
PERMANOVA
df = 1
R2 = 0.0851
p = 0.528
Group
FPD
Health
PCoA2 (14%)
0.6
0.3
0.0
−0.3
−0.5
0.0
0.5
1.0
PCoA1 (30%)
FPD Health
D
Relative abundance %
100
80
60
40
20
0
FPD
Health
Others
Spirochaetota
Verrucomicrobiota
Campylobacterota
Deferribacterota
Fusobacteriota
Actinomycetota
Pseudomonadota
Thermodesulfobacteriota
Bacteroidota
Bacillota
E
Relative abundance %
100
80
60
40
20
0
FPD
Health
Others
Mediterraneibacter
Megamonas
Rikenellaceae_RC9_gut_group
UCG-005
Enterococcus
Faecalibacterium
Desulfovibrio
Streptococcus
Romboutsia
Bacteroides
F
FPD
g__Fusobacterium
f__Fusobacteriaceae
o__Fusobacteriales
c__Fusobacteriia
p__Fusobacteriota
−4
−3
−2
−1
0
1
2
3
4
LDA Scores (log10)
