# Supplementary material for: Integrated multi-omics reveals persistent extracellular matrix remodeling in chronic duck footpad dermatitis
Source: Poult Sci. 2026 Sep 23;105(12):107830. doi: 10.1016/j.psj.2026.107830 (PMC13636073; doi:10.1016/j.psj.2026.107830)
Supplement: Supplementary file 5 [file mmc5.pdf]

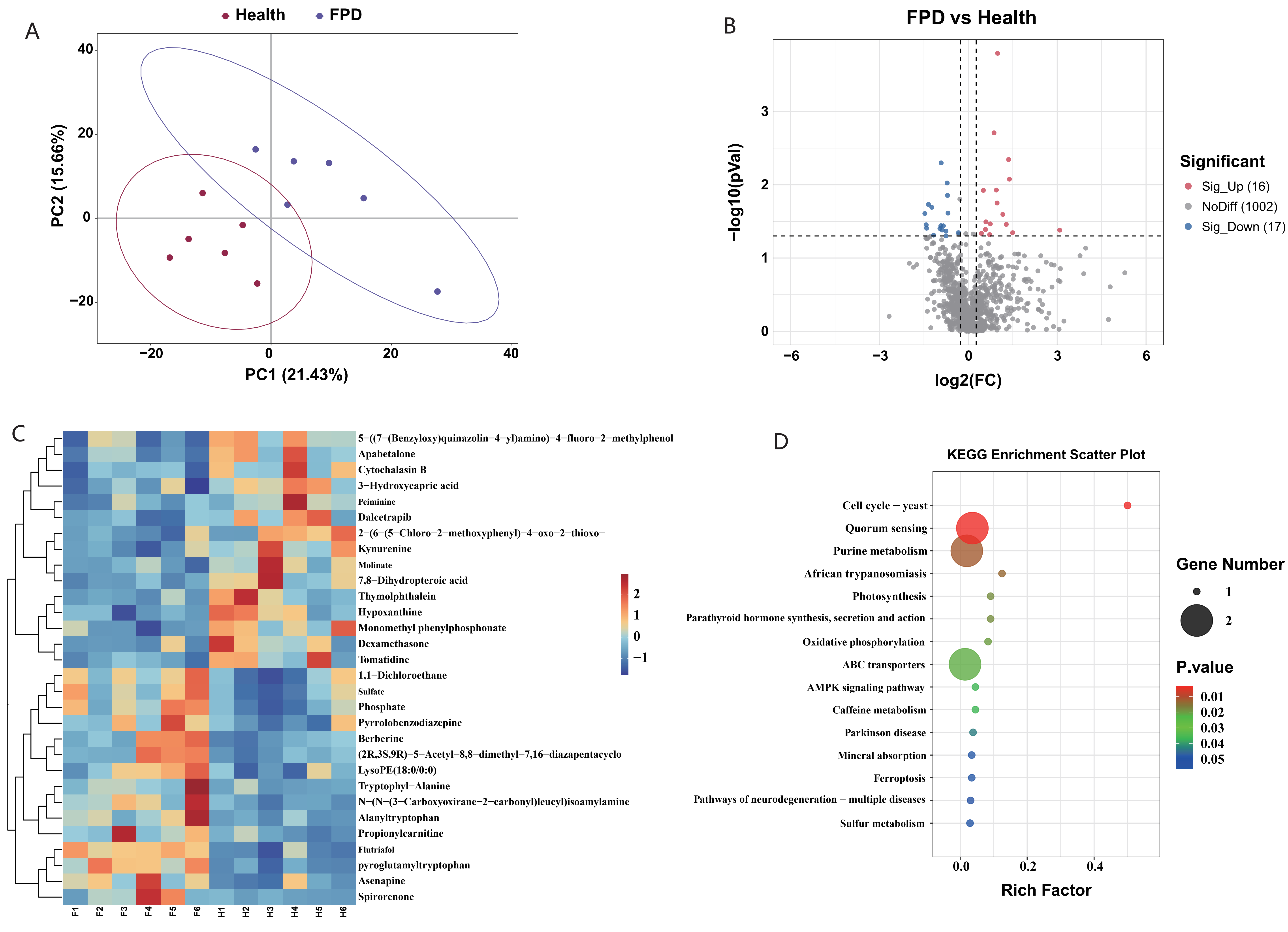
A
Health
FPD
PC2 (15.66%)
PC1 (21.43%)
40
20
0
−20
−20
0
20
40
B
FPD vs Health
−log10(pVal)
log2(FC)
3
2
1
0
−6
−3
0
3
6
Significant
Sig_Up (16)
NoDiff (1002)
Sig_Down (17)
C
5−((7−(Benzyloxy)quinazolin−4−yl)amino)−4−fluoro−2−methylphenol
Apabetalone
Cytochalasin B
3−Hydroxycapric acid
Peiminine
Dalcetrapib
2−(6−(5−Chloro−2−methoxyphenyl)−4−oxo−2−thioxo−
Kynurenine
Molinate
7,8−Dihydropteroic acid
Thymolphthalein
Hypoxanthine
Monomethyl phenylphosphonate
Dexamethasone
Tomatidine
1,1−Dichloroethane
Sulfate
Phosphate
Pyrrolobenzodiazepine
Berberine
(2R,3S,9R)−5−Acetyl−8,8−dimethyl−7,16−diazapentacyclo
LysoPE(18:0/0:0)
Tryptophyl−Alanine
N−(N−(3−Carboxyoxirane−2−carbonyl)leucyl)isoamylamine
Alanyltryptophan
Propionylcarnitine
Flutriafol
pyroglutamyltryptophan
Asenapine
Spirorenone
2
1
0
−1
F1
F2
F3
F4
F5
F6
H1
H2
H3
H4
H5
H6
D
KEGG Enrichment Scatter Plot
Cell cycle − yeast
Quorum sensing
Purine metabolism
African trypanosomiasis
Photosynthesis
Parathyroid hormone synthesis, secretion and action
Oxidative phosphorylation
ABC transporters
AMPK signaling pathway
Caffeine metabolism
Parkinson disease
Mineral absorption
Ferroptosis
Pathways of neurodegeneration − multiple diseases
Sulfur metabolism
0.0
0.2
0.4
Rich Factor
Gene Number
1
2
P.value
0.01
0.02
0.03
0.04
0.05
