# Supplementary material for: Integrated multi-omics reveals persistent extracellular matrix remodeling in chronic duck footpad dermatitis
Source: Poult Sci. 2026 Sep 23;105(12):107830. doi: 10.1016/j.psj.2026.107830 (PMC13636073; doi:10.1016/j.psj.2026.107830)
Supplement: Supplementary file 6 [file mmc6.pdf]

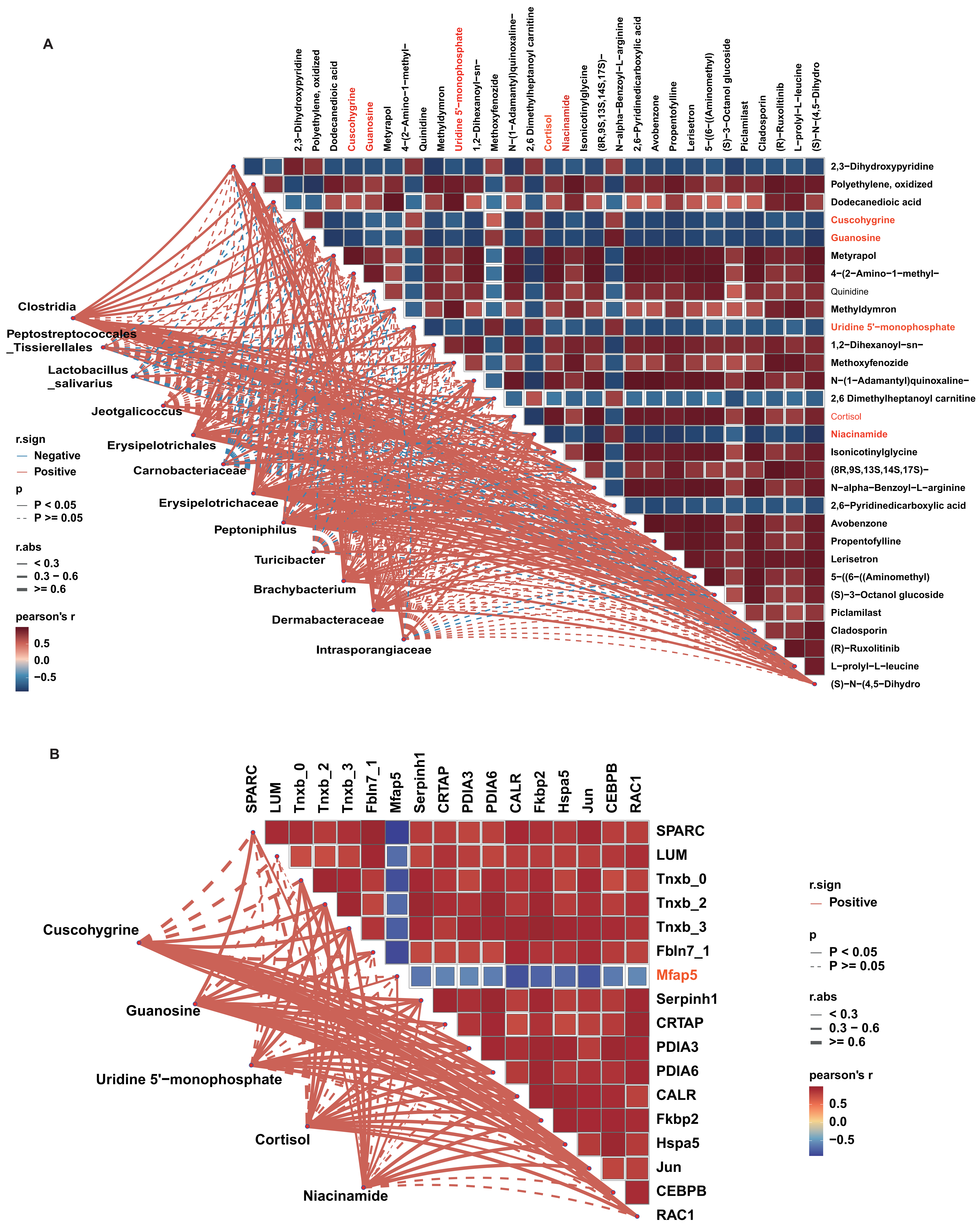
A
2,3−Dihydroxypyridine
Polyethylene, oxidized
Dodecanedioic acid
Cuscohygrine
Guanosine
Metyrapol
4−(2−Amino−1−methyl−
Quinidine
Methyldymron
Uridine 5'−monophosphate
1,2−Dihexanoyl−sn−
Methoxyfenozide
N−(1−Adamantyl)quinoxaline−
2,6 Dimethylheptanoyl carnitine
Cortisol
Niacinamide
Isonicotinylglycine
(8R,9S,13S,14S,17S)−
N−alpha−Benzoyl−L−arginine
2,6−Pyridinedicarboxylic acid
Avobenzone
Propentofylline
Lerisetron
5−((6−((Aminomethyl)
(S)−3−Octanol glucoside
Piclamilast
Cladosporin
(R)−Ruxolitinib
L−prolyl−L−leucine
(S)−N−(4,5−Dihydro
Clostridia
Peptostreptococcales _Tissierellales
Lactobacillus _salivarius
Jeotgalicoccus
Erysipelotrichales
Carnobacteriaceae
Erysipelotrichaceae
Peptoniphilus
Turicibacter
Brachybacterium
Dermabacteraceae
Intrasporangiaceae
r.sign
Negative
Positive
p
P < 0.05
P >= 0.05
r.abs
< 0.3
0.3 − 0.6
>= 0.6
pearson's r
0.5
0.0
−0.5
B
SPARC
LUM
Tnxb_0
Tnxb_2
Tnxb_3
Fbln7_1
Mfap5
Serpinh1
CRTAP
PDIA3
PDIA6
CALR
Fkbp2
Hspa5
Jun
CEBPB
RAC1
Cuscohygrine
Guanosine
Uridine 5'−monophosphate
Cortisol
Niacinamide
r.sign
Positive
p
P < 0.05
P >= 0.05
r.abs
< 0.3
0.3 − 0.6
>= 0.6
pearson's r
0.5
0.0
−0.5
