# Supplementary material for: Integrated multi-omics reveals persistent extracellular matrix remodeling in chronic duck footpad dermatitis
Source: Poult Sci. 2026 Sep 23;105(12):107830. doi: 10.1016/j.psj.2026.107830 (PMC13636073; doi:10.1016/j.psj.2026.107830)
Supplement: Supplementary file 7 [file mmc7.docx]

**Integrated multi-omics reveals persistent extracellular matrix remodeling in chronic duck footpad dermatitis**

Cui Ma^a,b^, Yunhe Zong^a,b^, Hao Zhang^a,b^, Zhenhua Liang^a,b^, Ailuan Pan^a,b^, Jinsong Pi^a,b^^[1]^*, Yan Wu^a,b^^[2]^*

^a^ Institute of Animal Husbandry and Veterinary Science, Hubei Academy of Agricultural Sciences, Wuhan, 430064, China.

^b^ Hubei Key Laboratory of Animal Embryo Engineering and Molecular Breeding, Wuhan, 430064, China.

* Corresponding Author: Yan Wu, E-mail: [wuyanwh@163.com](mailto:wuyanwh@163.com); Jinsong Pi, E-mail: [pijinsong@sina.com](mailto:pijinsong@sina.com).

**Supplementary Figures**

**Figure S1 Transcriptomic analysis of duck footpad tissues with footpad dermatitis (FPD).** (A) Hierarchical clustering heatmap of global gene expression profiles. (B) Relative mRNA expression levels of representative integrin subunits (ITGB1, ITGB3, ITGB6, and ITGB8). (C) Representative immunohistochemical staining of ITGB1 in footpad tissues. Scale bar = 100 μm. (D) Kyoto Encyclopedia of Genes and Genomes (KEGG) pathway enrichment analysis of differentially expressed genes (DEGs). (E) Gene dendrogram generated by weighted gene co-expression network analysis (WGCNA), showing the identified co-expression modules. (F) Module–trait relationship heatmap showing correlations between WGCNA modules and FPD status. Health, healthy ducks; FPD, ducks with footpad dermatitis. n = 3 biological replicates per group. Data in (B) are presented as mean ± SEM, and differences between groups were analyzed using an unpaired two-tailed Student's t-test. *P* < 0.05 was considered statistically significant.

**Figure S2 Proteomic analysis of duck footpad tissues with footpad dermatitis (FPD).** (A) Hierarchical clustering heatmap of differentially abundant proteins (DAPs) in the Health and FPD groups. DAPs were identified using *P* < 0.05 and fold change > 1.2 or < 0.83. (B) Kyoto Encyclopedia of Genes and Genomes (KEGG) pathway enrichment analysis of DAPs. Health, healthy ducks; FPD, ducks with footpad dermatitis. n = 4 biological replicates per group.

**Figure S3 Footpad microbiota composition and predicted functional profiles in duck footpads with footpad dermatitis (FPD).** (A) Alpha diversity based on the ACE index. (B) Alpha diversity based on the Simpson index. (C) Principal component analysis (PCA) of footpad microbial communities in the Health and FPD groups. (D) Relative abundance of the ten most abundant bacterial genera. (E) Predicted microbial functional profiles inferred using PICRUSt2 based on 16S rRNA gene sequencing data. Health, healthy ducks; FPD, ducks with footpad dermatitis. n = 8 biological replicates per group.

**Figure S4 Gut microbiota composition in ducks with footpad dermatitis (FPD).** (A) Alpha diversity based on the Chao1 index. (B) Alpha diversity based on the Simpson index. (C) Principal coordinates analysis (PCoA) based on Bray–Curtis dissimilarity. Group differences in microbial community composition were evaluated using permutational multivariate analysis of variance (PERMANOVA). (D) Relative abundance of dominant bacterial phyla. (E) Relative abundance of dominant bacterial genera. (F) Linear discriminant analysis effect size (LEfSe) analysis of differentially abundant bacterial taxa using a linear discriminant analysis (LDA) score > 3. Health, healthy ducks; FPD, ducks with footpad dermatitis. n = 6 biological replicates per group.

**Figure S5 Serum metabolomic analysis in ducks with footpad dermatitis (FPD).** (A) Principal component analysis (PCA) of serum metabolomic profiles. (B) Volcano plot of differential serum metabolites. Differential metabolites were identified using *P* < 0.05, fold change > 1.2, and variable importance in projection (VIP) > 1. (C) Hierarchical clustering heatmap of representative differential serum metabolites. (D) Kyoto Encyclopedia of Genes and Genomes (KEGG) pathway enrichment analysis of differential metabolites. Health, healthy ducks; FPD, ducks with footpad dermatitis. n = 6 biological replicates per group.

**Figure S6 Cross-omics correlation analysis of microbial, metabolic, and host molecular alterations in duck footpads with footpad dermatitis (FPD).** (A) Correlation network of representative microbial taxa and differential metabolites. (B) Correlation network of representative differential metabolites and extracellular matrix (ECM)-related proteins involved in mechanotransduction and extracellular matrix remodeling. Nodes represent microbial taxa, metabolites, or proteins, and edges represent correlations between variables. Red and blue lines indicate positive and negative correlations, respectively. Correlations were assessed using Pearson's correlation analysis. The correlations shown represent statistical associations and do not imply causal relationships.

1. * Correspondence: Dr. Yan Wu, [wuyanwh@163.com](mailto:wuyanwh@163.com)

   * Correspondence: Dr. Jinsong Pi, [pijinsong@sina.com](mailto:pijinsong@sina.com)
